# Effect of physical activity on prevention of postpartum depression: A dose-response meta-analysis of 186,412 women

**DOI:** 10.3389/fpsyt.2022.984677

**Published:** 2022-11-04

**Authors:** Mengqi Yuan, Hongyang Chen, Dongmei Chen, Donggui Wan, Fan Luo, Chenyang Zhang, Yunxin Nan, Xiaoning Bi, Jing Liang

**Affiliations:** ^1^Graduate School, Beijing University of Chinese Medicine, Beijing, China; ^2^Department of Oncology, China-Japan Friendship Hospital, Beijing, China; ^3^Department of Obstetrics and Gynecology, China-Japan Friendship Hospital, Beijing, China

**Keywords:** physical activity, postpartum depression, mental-health, dose-response analysis, meta-analysis

## Abstract

**Background:**

Physical activity (PA) is considered a favorable preventive intervention for postpartum depression (PPD), but evidence defining a corresponding dose-response relationship is lacking. This meta-analysis was conducted to assess the protective effects of PA on PPD and define a potential dose-response relationship between them.

**Methods:**

PubMed, Medline, Embase, and Web of Science were searched from 1968 to May 2022. Only randomized control trials (RCTs) and prospective studies were considered, and the PICOS tool was used to identify eligible articles based on the inclusion and exclusion criteria. Effect-size estimates were unified as odds ratio (OR) and 95% confidence interval (CI). We calculated the ORs and their 95% CI for studies that did not report them using the Practical Meta-Analysis Effect Size Calculator.

**Results:**

A total of 23 studies were eligible, including 14 RCTs and 9 prospective cohort studies. The overall analysis showed a statistically significant positive association between PA and PPD prevention (adjusted OR = 0.73; 95% CI: 0.61–0.87; *P* < 0.001). Subgroup analyses indicated that studies conducted in Europe demonstrated a significant correlation between PA and reduced PPD risk (adjusted OR = 0.85, 95% CI: 0.76–0.95, *P* = 0.004). Concerning PA type, sports activity was associated with relieving PPD symptoms (adjusted OR = 0.89, 95% CI: 0.78 to 1.00, *P* < 0.001), while work (adjusted OR = 1.05, 95% CI: 0.37–2.97, *P* = 0.065) and household activities (adjusted OR = 1.16, 95% CI: 0.89–1.52, *P* = 0.986) contributed to a greater risk of PPD. Our dose-response analysis revealed a reverse J-shaped trend between ascending PA duration and PPD incidence.

**Conclusion:**

This meta-analysis identified PA as a potential intervention to reduce the risk of PPD. The dose-response analysis revealed that at least 90 min of PA per week could efficiently decrease the risk of PPD.

**Systematic review registration:**

https://www.crd.york.ac.uk/PROSPERO/, identifier: CRD42022335731.

## Introduction

Postpartum depression (PPD) is a specific type of mental disorder that might occur up to 1 year after giving birth and affects 5–25% of new mothers (1). However, ~50% of the affected mothers remain undiagnosed by healthcare professionals (2). As a severe mental health issue that may severely affect women and their families worldwide, PPD might disrupt the mother-infant relationship and negatively impact the child's cognitive, behavioral, and social-emotional development in the long term (3–5). In severe cases, women with PPD may develop suicidal tendencies and even harm their babies (6–8). Many causes have been attributed to PPD, including physiological, situational, or social difficulties (9). The major predisposing factors include stressful life events, child-care stress, prenatal anxiety, prior episodes of PPD (10), marital conflicts, and single-parent households (11).

At present, among the many interventions recommended for treating PPD are antidepressant medications, cognitive behavioral therapy (CBT), psychosocial support through support groups, electroconvulsive therapies, and complementary therapy (11, 12). However, antidepressants might affect the infants' health *via* breastfeeding, and the other three therapies are usually expensive, time-consuming, and require a lot of energy that might not be at the disposal of the suffering mother. Thus, newer and more efficient primary PPD prevention strategies are required to improve the recovery process of these patients.

Complementary therapy has been recognized as an effective method for the prevention and treatment of chronic diseases (13). *Nigella sativa*, a kind of herb, is helpful in the treatment of asthma, liver and kidney disease, influenza, and gastrointestinal problems (14). Phytosterols, a group of natural compounds of plant-cell membranes, could be easily obtained from the diet and have been proven to decrease the risk of chronic diseases, such as cardiovascular diseases, obesity, and diabetes (15). Like other complementary therapies, physical activities (PA) have an advantage in improving the conditions of many chronic diseases, such as cognitive impairment, coronary artery disease, diabetes, and depression (16, 17).

The three main categories of PA during pregnancy include (1) physical exercise, a subset of planned, structured, and repetitive PA, such as aerobic exercise, stretching and breathing exercises, walking programs, strength exercises, pilates, and yoga; (2) housework PA, such as child care and household chores; and (3) work activities. *The National Physical Activity Guidelines for Americans*, released in 2008, encourages women to participate in 150–300 min of moderate-intensity aerobic activities per week during pregnancy and after delivery (18). In addition, the *American College of Obstetricians and Gynecologists (ACOG)* recommends 20–30 min of daily moderate-intensity PA to stay healthy during pregnancy (19). In 2014, a randomized controlled trial (RCT) conducted in America reported that 90 min of yoga per week could help reduce the risk of PPD (20), and in 2019, RCTs performed in Spain suggested that 180 min of exercise per week were needed to prevent PPD (21, 22). In contrast, a study from Northern Taiwan showed that depressive symptoms were not affected by the duration or frequency of PA (23). Thus, the optimal duration of PA that could help reduce the risk of PPD remains undetermined.

In this study, we conducted a meta-analysis to determine the effects of PA on PPD and to define the dose-response relationship between them to provide more detailed evidence for health authorities and pregnant women to reduce the risk of PPD.

## Methods

### Registration and reporting format

This meta-analysis was conducted following the guidelines of the Preferred Reporting Items for Systematic Reviews and Meta-analyses (PRISMA) statement (24) and the Meta-analysis of Observational Studies in Epidemiology (MOOSE) statement (25). Both the PRISMA and MOOSE checklists used in this study are shown in Supplementary Table 1. The study protocol was registered in the International Prospective Register of Systematic Reviews (PROSPERO) (registration number: CRD42022335731).

### Search strategy

A literature search was performed from 1968 to May 2022 on PubMed, Medline, Embase, and Web of Science databases. The PICOS principle of this meta-analysis was as follows: (P) Population involving women who were pregnant or up to 1 year postpartum; (I) Intervention involving all kinds of physical activities; (C) Comparator including standard prenatal and postpartum care; (O) Outcomes dicussing the effect of PA on PPD symptoms; and (S) Study type including randomized controlled trials (RCTs) and prospective cohort studies. The detailed search terms are presented in the Supplementary Table 2.

### Eligibility criteria

Qualified articles were restricted to the following criteria: (1) study participants including women who were pregnant or up to 1 year postpartum; (2) endpoints including the effects of PA on PPD symptoms, identified *via* a structured clinical interview and a validated tool [e.g., Edinburgh Postnatal Depression Scale, EPDS (26)] or judged by a healthcare professional (e.g., general practitioner or psychiatrist); (3) study type including RCTs and prospective cohort studies; (4) intervention including any type of PA during or after pregnancy; and (5) language including studies published in English. Studies were excluded if they (1) did not contain data of interest for this meta-analysis; (2) were duplicated publications; (3) were reviews, case reports, or comments; or (4) contained animal or genomics experiments.

### Study selection

Endnote version 20 was used for literature management to file the literature search records. The study selection process, conducted by two authors (M. Y. and H. C.), was performed following these three steps: First, two authors screened the titles of all the recovered articles and removed duplicates. An article was included if at least one reviewer judged it qualified. If there were any doubts about the studies, they were included in the abstract review phase. Second, the selected studies were filtrated by reviewing their abstracts and reevaluated by the two independent reviewers according to the inclusion and exclusion criteria. Finally, using standardized eligibility criteria, the two independent reviewers assessed the full texts of potentially relevant studies. If the reviewers disagreed on any of the three steps, a third independent reviewer from our group intervened to make a judgment after mutual discussions.

### Data extraction

The two reviewers (M. Y. and H. C) separately extracted the following data from the eligible studies: first author, year of publication, the country where the study was conducted, gender, sample size, study type, age of the population, PA type, frequency and duration of PA, PA intensity, and assessment of PPD.

### Risk of bias in individual studies

The two authors independently evaluated all eligible studies for bias using the Risk of Bias in Non-Randomized Studies of Interventions (ROBINS-I) assessment tool (27), which contained seven different types of bias and divided each part into five grades of risk of bias (ROB). Eligible articles were classified into three levels based on the number of components for which high ROB potentially existed, namely, high risk (five or more), moderate risk (three or four), and low risk (two or less).

### Statistical analyses

STATA software version 14.1 for Windows (Stata Corp, College Station, TX, USA) was used for data management and analysis. A two-sided *P*-value of < 0.05 was considered significantly different. The effect size of this meta-analysis was odds ratio (OR). For the studies that did not report ORs, we calculated their ORs and corresponding 95% confidence intervals (95% CI) using the Practical Meta-Analysis Effect Size Calculator (28). Generalized least squares regression (29, 30), which was proposed by Greenland and Longnecker, was used to estimate the dose-response relationship by summarizing dose-response data. Furthermore, the nonlinearity test between PA duration and risk of PPD was calculated using restricted cubic splines of exposure distribution with 3 knots (25, 50, and 75th percentiles).

The inconsistency index (*I*^2^) statistic was used to quantify potential heterogeneity among the studies. Obvious heterogeneity was determined for *I*^2^ ≥50%. Publication bias was assessed using Begg's funnel plots and Egger regression asymmetry tests. The trim-and-fill method was applied to reckon the number of missing studies in theory. Sensitivity analysis was performed to judge the solidity of the overall results by consecutively omitting the study.

To clarify the possible causes of between-study heterogeneity from clinical and methodological perspectives, prespecified subgroup analyses were performed based on the study region, the age of the participants, gestation age of the pregnant participants, activity duration for a week, PA types, PA intensity, PA timing, and study type.

## Results

### Eligible studies

**Figure 1:**
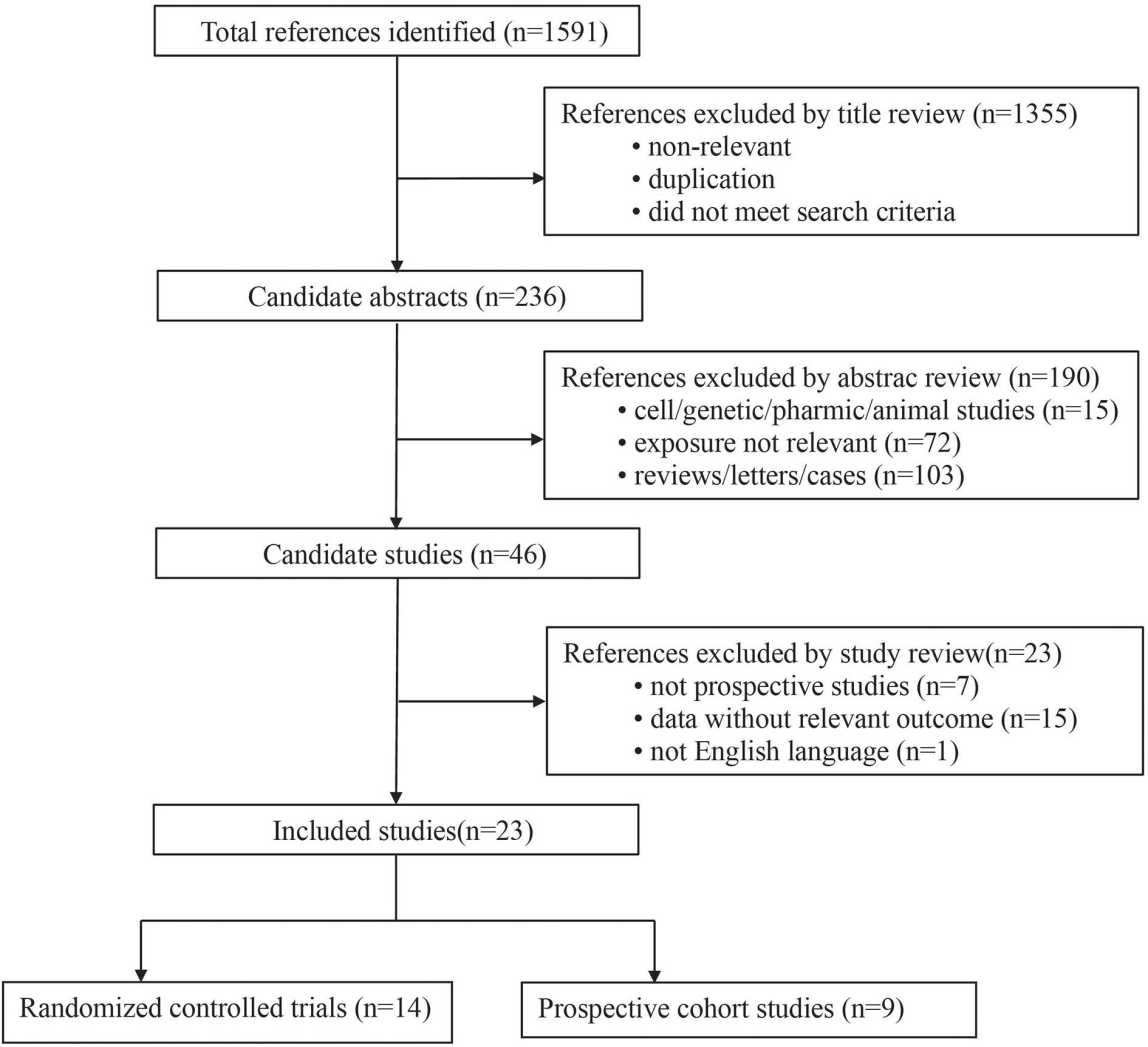
Flow chart of records retrieved, screened, and included in this meta-analysis.

A total of 1,591 articles were initially retrieved after screening the public database using the predefined retrieval code. Of them, 23 (14 RCTs and 9 prospective cohort studies) met the inclusion criteria of this study, comprising a total of 186,412 subjects. The detailed selection process is presented in Figure 1.

### Study characteristics

The baseline characteristics of the 23 (20–22, 28, 31–49) eligible studies are displayed in Table 1. Of them, 12 were conducted in Europe (21, 33, 36–38, 40, 41, 44, 45, 47, 48), 6 in America (20, 32, 34, 42, 43, 46), and 5 in Asia (28, 31, 35, 39, 49). Of the 14 RCT studies (20–22, 28, 31–40), 4,607 participants with 1,722 cases were selected in the meta-analysis. Among the 9 prospective cohort studies (41–49), 181,805 participants with 43,069 cases were analyzed.

**Table 1:** Baseline characteristics of the studies included in this meta-analysis.

**Year**	**Author**	**Locat** **ion**	**Study type**	**Age (years)**	**Gestat ional** **age (weeks)**	**Total numb** **er**	**Numb** **er of** **case**	**Numb** **er of cont** **rol**	**PA type**	**Frequ** **ency** **(min)** **of PA**	**Dura** **tion** **(week^−1^)** **of PA**	**Inten** **sity**	**PPD asses** **sment tool**	**Time of PPD asses** **sment**	**Pregn** **ancy peri** **od of PA**	**Mean case**	**Mean cont** **rol**	**SD case**	**SD cont** **rol**	**OR** **(95%** **CI)**
2008	Heh	China	RCT	20–35	38–42	63	33	30	Stretching exercise, warm-up and cool-down period of exercise	60	3	NA	EPDS	20 weeks postpartum	4weeks postpartum	10.2	12.7	3.6	3.9	NA
2008	Ko	China	RCT	34.3	NA	61	31	30	Pilates, yoga	60	3	Low	CES-D	3 weeks postpartum	NA	12.42	14.53	5.37	6.94	NA
2009	Da Costa	Canada	RCT	34.3	NA	88	46	42	Aerobic exercise, stretching, and strength exercises	30	3	Moderate to high	EPDS	NA	4–48 weeks postpartum	13.6	13.6	3.6	3.9	NA
2009	Da Costa	Canada	RCT	34.3	NA	88	46	42	Aerobic exercise, stretching, and strength exercises	30	3	Moderate to high	EPDS	12 weeks postpartum	4–48 weeks postpartum	8.6	9	3.8	3.8	NA
2009	Da Costa	Canada	RCT	34.3	NA	88	46	42	Aerobic exercise, stretching, and strength exercises	30	3	Moderate to high	EPDS	24 weeks postpartum	4–48 weeks postpartum	7.4	7.6	3.7	3.7	NA
2012	SONGØY GARD	Norway	RCT	30.6	20–36	719	379	340	Aerobic activity, endurance training, and strength/balance exercises	60	12	Moderate to high	EPDS	12 weeks postpartum	2–3 pregnancy trimesters	NA	NA	NA	NA	0.73 (0.4–1.5)
2012	SONGØY GARD	Norway	RCT	30.6	20–36	719	379	340	Aerobic activity, endurance training, and strength/balance exercises	60	12	Moderate to high	EPDS	12 weeks postpartum	2–3 pregnancy trimesters	NA	NA	NA	NA	0.44 (0.1–1.5)
2014	Bersha dsky	USA	RCT	NA	12–19	51	38	13	Prenatal Hatha yoga	90	1	Low to moderate	CES-D	8 weeks postpartum	1–3 pregnancy trimesters	3.92	6.63	2.68	2.83	NA
2015	Mohamm adi	Iran	RCT	25.3	26–32	127	43	42	Home-based stretching and breathing exercises	20–30	3	Low	EPDS	26–32 weeks of gestation	1–3 pregnancy trimesters	7.77	8.14	3.86	3.94	NA
2015	Mohamm adi	Iran	RCT	25.3	26–32	127	43	42	Home-based stretching and breathing exercises	20–30	3	Low	EPDS	4 weeks postpartum	1–3 pregnancy trimester	7.66	7.46	5.46	4.5	NA
2015	Mohammadi	Iran	RCT	25.3	26–32	127	43	42	Home-based stretching and breathing exercises	20–30	3	Low	EPDS	8 weeks postpartum	1–3 pregnancy trimesters	6.58	6.5	6	5.5	NA
2015	Buttner	USA	RCT	18–45	NA	57	28	29	Yoga	60	1	NA	HDRS	Pre-treatment, 2 weeks, 4 weeks, 6 weeks, and 8 weeks (post-treatment)	≥ 6 weeks postpartum	5.87	8.52	6.03	5.43	NA
2018	Daley	UK	RCT	16–50	10–24	784	146	133	Brisk walk	30	5	NA	EPDS	24 weeks postpartum	1–2 pregnancy trimesters	6.8	6.6	4.8	4.7	1.07 (0.36–0.17)
2019	Aguilar	Spain	RCT	21–43	NA	129	64	65	Warm-up, aerobic and strength, and endurance exercises in an aquatic environment	60	3	Moderate	EPDS	6 weeks postpartum	2–3 pregnancy trimesters	6.41	10.17	3.68	2.38	NA
2019	Vargas-Terrones	Spain	RCT	NA	12–16	124	70	54	Walking, stretching, aerobic exercise, muscular strength, coordination and balance, floor exercises, relaxation	60	3	Moderate	CES-D	6 weeks postpartum	1–3 pregnancy trimesters	11	10.06	7.7	6.8	NA
2019	Coll	Brazil	RCT	27.1	16.5	639	213	426	Warm–up exercise, aerobic activity, strength and floor exercises, and passive and active stretching exercises	60	1	Moderate	EPDS	12 weeks postpartum	2–3 pregnancy trimesters	4.8	5.4	3.7	4.1	NA
2020	Özkan	Turkey	RCT	28.9	38–42	65	34	31	Exercise	30	5	Low to moderate	EPDS	4 weeks postpartum	Postpartum	7.29	12.54	1.67	2.65	NA
2021	Ana	India	RCT	18–45	14–36	1406	449	957	Exercise, other hobbies, household chores, sedentary activities, and other everyday daily activities.	NA	NA	Low to moderate	EPDS	1 weeks postpartum	2–3 pregnancy trimesters	NA	NA	NA	NA	3.15 (1.98–0.02)
2021	Navas	Switzerland	RCT	18–40	14–20	294	148	146	Water aerobics exercise	45	3	Low	EPDS	20 weeks postpartum	1–2 pregnancy trimesters	NA	NA	NA	NA	0.23 (0.05–0.11)
2009	Strom	Denmark	Prospective cohort	25–40	NA	70866	26494	44372	Leisure	NA	NA	Moderate	Admission to hospital with diagnosis of a depressive episode antidepressant medication prescription	1–52 weeks postpartum	1–3 pregnancy trimesters	NA	NA	NA	NA	1.20 (0.83–0.72)
2009	Strom	Denmark	Prospective cohort	25–40	NA	70866	26494	44372	Leisure	NA	NA	High		1–52 weeks postpartum	1–3 pregnancy trimesters	NA	NA	NA	NA	0.88 (0.52–0.52)
2009	Strom	Denmark	Prospective cohort	25–40	NA	70866	26494	44372	Leisure	NA	NA	Moderate to high		1–52 weeks postpartum	1–3 pregnancy trimesters	NA	NA	NA	NA	1.01 (0.79–0.53)
2009	Strom	Denmark	Prospective cohort	25–40	NA	70866	26494	44372	Leisure	NA	NA	Moderate	Outpatient contact with diagnosis of a depressive episode antidepressant medication prescription	1–52 weeks postpartum	1–3 pregnancy trimesters	NA	NA	NA	NA	0.90 (0.79–0.02)
2009	Strom	Denmark	Prospective cohort	25–40	NA	70866	26494	44372	Leisure	NA	NA	High		1–52 weeks postpartum	1–3 pregnancy trimesters	NA	NA	NA	NA	0.94 (0.82–0.08)
2009	Strom	Denmark	Prospective cohort	25–40	NA	70866	26494	44372	Leisure	NA	NA	Moderate to high		1–52 weeks postpartum	1–3 pregnancy trimesters	NA	NA	NA	NA	0.81 (0.66–0.99)
2011	Demissie	USA	Prospective cohort	24–35	NA	550	374	176	Total activity	NA	NA	NA	EPDS	NA	12 months postpartum	NA	NA	NA	NA	1.39 (0.86–0.28)
2011	Demissie	USA	Prospective cohort	24–35	NA	550	374	176	Work	NA	NA	NA	EPDS	NA	12 months postpartum	NA	NA	NA	NA	1.73 (1.01–0.97)
2011	Demissie	USA	Prospective cohort	24–35	NA	550	374	176	Receational	NA	NA	NA	EPDS	NA	12 months postpartum	NA	NA	NA	NA	1.39 (0.89–0.17)
2011	Demissie	USA	Prospective cohort	24–35	NA	550	374	176	Child and adult care	NA	NA	NA	EPDS	NA	12 months postpartum	NA	NA	NA	NA	1.42 (0.91–0.22)
2011	Demissie	USA	Prospective cohort	24–35	NA	550	374	176	Indoor household	NA	NA	NA	EPDS	NA	12 months postpartum	NA	NA	NA	NA	1.36 (0.86–0.04)
2011	Demissie	USA	Prospective cohort	24–35	NA	550	374	176	Outdoor household	NA	NA	NA	EPDS	NA	12 months postpartum	NA	NA	NA	NA	0.89 (0.32–0.45)
2013	Demissie	USA	Prospective cohort	30	17–22	652	432	220	Total activity	60	1	Moderate to high	EPDS	12 weeks postpartum	2–3 pregnancy trimesters	NA	NA	NA	NA	1.07 (0.46–0.46)
2013	Demissie	USA	Prospective cohort	30	17–22	652	432	220	Work	60	1	Moderate to high	EPDS	12 weeks postpartum	2–3 pregnancy trimesters	NA	NA	NA	NA	0.14 (0.02–0.17)
2013	Demissie	USA	Prospective cohort	30	17–22	652	432	220	Recreational	60	1	Moderate to high	EPDS	12 weeks postpartum	2–3 pregnancy trimesters	NA	NA	NA	NA	0.84 (0.39–0.84)
2013	Demissie	USA	Prospective cohort	30	17–22	652	432	220	Child care	60	1	Moderate to high	EPDS	12 weeks postpartum	2–3 pregnancy trimesters	NA	NA	NA	NA	0.90 (0.36–0.84)
2013	Demissie	USA	Prospective cohort	30	17–22	652	432	220	Indoor household	60	1	Moderate to high	EPDS	12 weeks postpartum	2–3 pregnancy trimesters	NA	NA	NA	NA	1.19 (0.49–0.88)
2013	Demissie	USA	Prospective cohort	30	17–22	652	432	220	Outdoor household	60	1	Moderate to high	EPDS	12 weeks postpartum	2–3 pregnancy trimesters	NA	NA	NA	NA	1.25 (0.34–0.58)
2013	Demissie	USA	Prospective cohort	30	17–22	652	432	220	Transportation	60	1	Moderate to high	EPDS	12 weeks postpartum	2–3 pregnancy trimesters	NA	NA	NA	NA	0.95 (0.26–0.51)
2013	Demissie	USA	Prospective cohort	30	27–30	652	409	243	Total activity	60	1	Moderate to high	EPDS	12 weeks postpartum	2–3 pregnancy trimesters	NA	NA	NA	NA	1.02 (0.45–0.32)
2013	Demissie	USA	Prospective cohort	30	27–30	652	409	243	Work	60	1	Moderate to high	EPDS	12 weeks postpartum	2–3 pregnancy trimester	NA	NA	NA	NA	1.47 (0.50–0.33)
2013	Demissie	USA	Prospective cohort	30	27–30	652	409	243	Recreational	60	1	Moderate to high	EPDS	12 weeks postpartum	2–3 pregnancy trimesters	NA	NA	NA	NA	0.91 (0.4–2.07)
2013	Demissie	USA	Prospective cohort	30	27–30	652	409	243	Child care	60	1	Moderate to high	EPDS	12 weeks postpartum	2–3 pregnancy trimesters	NA	NA	NA	NA	1.33 (0.53–0.34)
2013	Demissie	USA	Prospective cohort	30	27–30	652	409	243	Indoor household	60	1	Moderate to high	EPDS	12 weeks postpartum	2–3 pregnancy trimesters	NA	NA	NA	NA	0.93 (0.39–0.25)
2013	Demissie	USA	Prospective cohort	30	27–30	652	409	243	Outdoor household	60	1	Moderate to high	EPDS	12 weeks postpartum	2–3 pregnancy trimesters	NA	NA	NA	NA	0.44 (0.05–0.15)
2013	Demissie	USA	Prospective cohort	30	27–30	652	409	243	Transportation	60	1	Moderate to high	EPDS	12 weeks postpartum	2–3 pregnancy trimesters	NA	NA	NA	NA	0.94 (0.26–0.43)
2018	Shakeel	Norway	Prospective cohort	30	NA	643	60	583	NA	1–74	1	Moderate to high	EPDS	12 weeks postpartum	1–3 pregnancy trimester	NA	NA	NA	NA	0.50 (0.22–0.17)
2018	Shakeel	Norway	Prospective cohort	30	NA	643	60	583	NA	75–149	1	Moderate to high	EPDS	12 weeks postpartum	1–3 pregnancy trimesters	NA	NA	NA	NA	0.50 (0.18–0.13)
2018	Shakeel	Norway	Prospective cohort	30	NA	643	60	583	NA	>150	1	Moderate to high	EPDS	12 weeks postpartum	1–3 pregnancy trimesters	NA	NA	NA	NA	0.20 (0.06–0.59)
2019	Van der Waerden	France	Prospective cohort	30	NA	15,538	7,859	7,935	Household/caregiving	90	NA	Low to high	EPDS	8 weeks postpartum	1–3 pregnancy trimesters	NA	NA	NA	NA	1.10 (1.01–0.19)
2019	Van der Waerden	France	Prospective cohort	30	NA	15,538	7,859	7,935	Occupational	90	NA	Low to high	EPDS	8 weeks postpartum	1–3 pregnancy trimesters	NA	NA	NA	NA	0.99 (0.91–0.08)
2019	Van der Waerden	France	Prospective cohort	30	NA	15,538	7,859	7,935	Transportation	90	NA	Low to high	EPDS	8 weeks postpartum	1–3 pregnancy trimesters	NA	NA	NA	NA	1.08 (1.00–0.15)
2019	Van der Waerden	France	Prospective cohort	30	NA	15,538	7,859	7,935	Leisure-time sedentary behavior	90	NA	Low to high	EPDS	8 weeks postpartum	1–3 pregnancy trimesters	NA	NA	NA	NA	1.16 (1.06–0.23)
2019	Van der Waerden	France	Prospective cohort	30	NA	15,538	7,859	7,935	Sports/exercise	90	NA	Low to high	EPDS	8 weeks postpartum	1–3 pregnancy trimesters	NA	NA	NA	NA	1.02 (0.93–0.09)
2019	Van der Waerden	France	Prospective cohort	30	NA	15,538	7,859	7,935	Total	90	NA	Low to high	EPDS	8 weeks postpartum	1–3 pregnancy trimesters	NA	NA	NA	NA	1.12 (1.03–0.19)
2020	Susukida	Japan	Prospective cohort	30	NA	92,743	4,734	NA	Exercise	NA	NA	low	EPDS	NA	NA	NA	NA	NA	NA	0.99 (0.94–0.04)
2020	Susukida	Japan	Prospective cohort	30	NA	92,743	376	NA	Exercise	NA	NA	Moderate	EPDS	NA	NA	NA	NA	NA	NA	0.97 (0.87–0.08)
2020	Susukida	Japan	Prospective cohort	30	NA	92,743	16	NA	Exercise	NA	NA	High	EPDS	NA	NA	NA	NA	NA	NA	1.25 (0.74–0.11)
2020	Susukida	Japan	Prospective cohort	30	NA	92,743	1,639	NA	Exercise	NA	NA	low to moderate	EPDS	NA	NA	NA	NA	NA	NA	1.07 (0.99–0.15)
2020	Susukida	Japan	Prospective cohort	30	NA	92,743	75	NA	Exercise	NA	NA	Low & high	EPDS	NA	NA	NA	NA	NA	NA	1.21 (0.97–0.51)
2020	Susukida	Japan	Prospective cohort	30	NA	92,743	30	NA	Exercise	NA	NA	moderate to high	EPDS	NA	NA	NA	NA	NA	NA	1.12 (0.79–0.57)
2020	Susukida	Japan	Prospective cohort	30	NA	92,743	300	NA	Exercise	NA	NA	Low to high	EPDS	NA	NA	NA	NA	NA	NA	1.42 (1.24–0.61)

PPD, postpartum depression; PA, physical activity; EPDS, Edinburgh Postnatal Depression Scale; CESD, Center for Epidemiologic Studies Depression Scale; HADS-D, Hospital Anxiety and Depression Scale; USA, the United States; UK, the United Kingdom; SD, standard deviation; OR, odds ratio; CI, confidence interval; NA, not available.

### ROB assessment

The items of the ROB assessment of each study are listed in Supplementary Table 3. The ROB rating was low in twenty-two studies and moderate in one study. Regarding confounding bias, 14 articles were determined as low risk, 8 as moderate risk, and one as high risk. An assessment of bias of the selection identified only one article as moderate risk but 22 as low risk. Bias resulting from deviation from the intended interventions was low in all studies, as well as bias in outcome measurement and selection.

### Overall analyses

**Figure 2:**
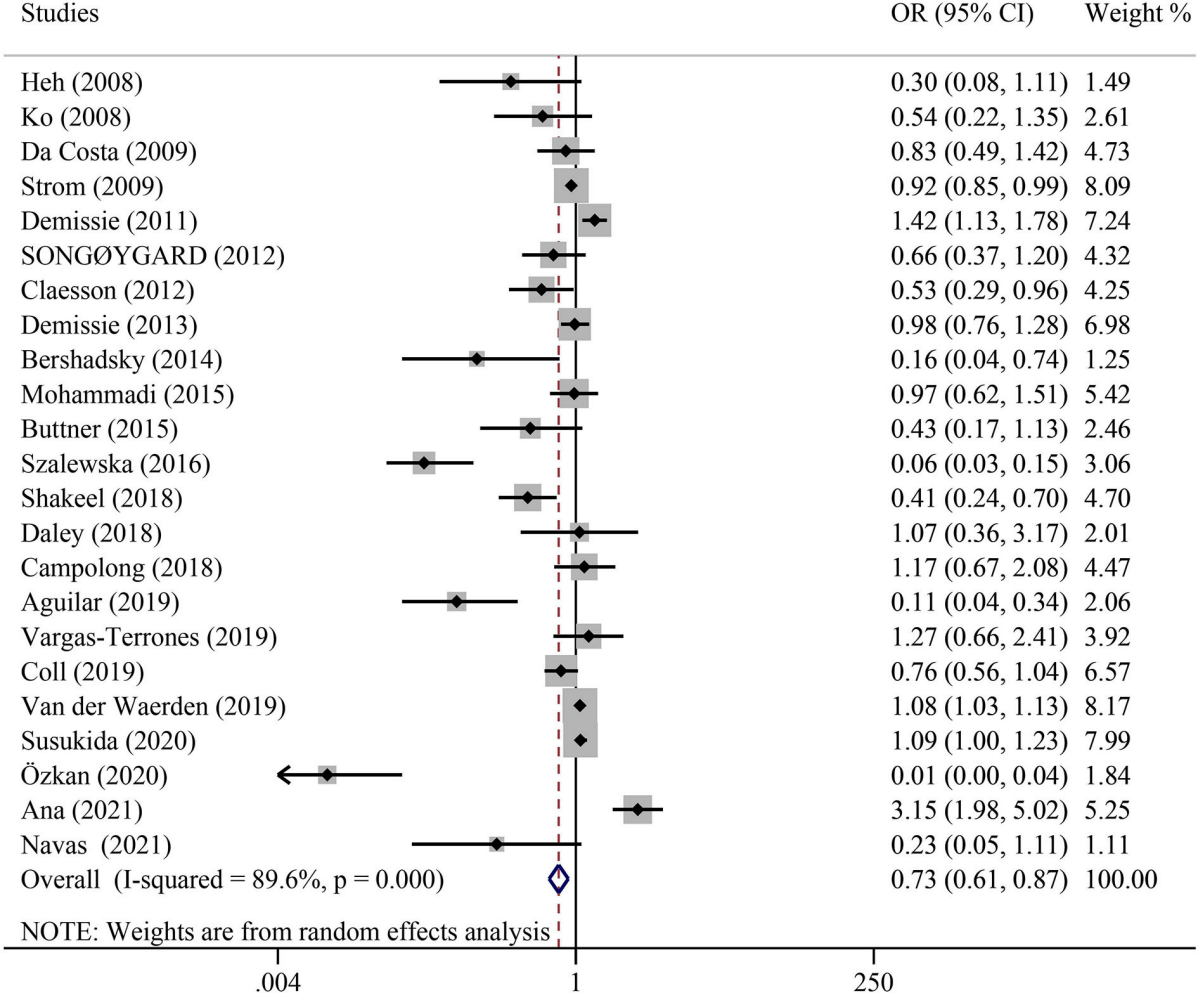
Overall analysis of the association between physical activity and postpartum depression with odds ratio (OR) and 95% confidence interval (CI).

When the results of all eligible articles (Table 2) were synthesized, we found a statistically significant positive association between PA and reduction of PPD risk (adjusted OR = 0.73; 95% CI: 0.61–0.87; *P* < 0.001) (Figure 2).

**Table 2:** The does-response analysis of physical activity duration and the risk of postpartum depression.

**Physical activity**	**OR (95% CI)**
**duration (minutes.week^−1^)**	
0	1.00
37.5	1.05 (0.96–1.14)
60	1.02 (0.93–1.13)
75	0.98 (0.90–1.07)
90	0.92 (0.85–1.00)
112	0.83 (0.75–0.91)
135	0.72 (0.61–0.85)
150	0.65 (0.52–0.82)
180	0.54 (0.38–0.77)

OR, odds ratio; CI, confidence interval.

### Publication bias

**Figure 3:**
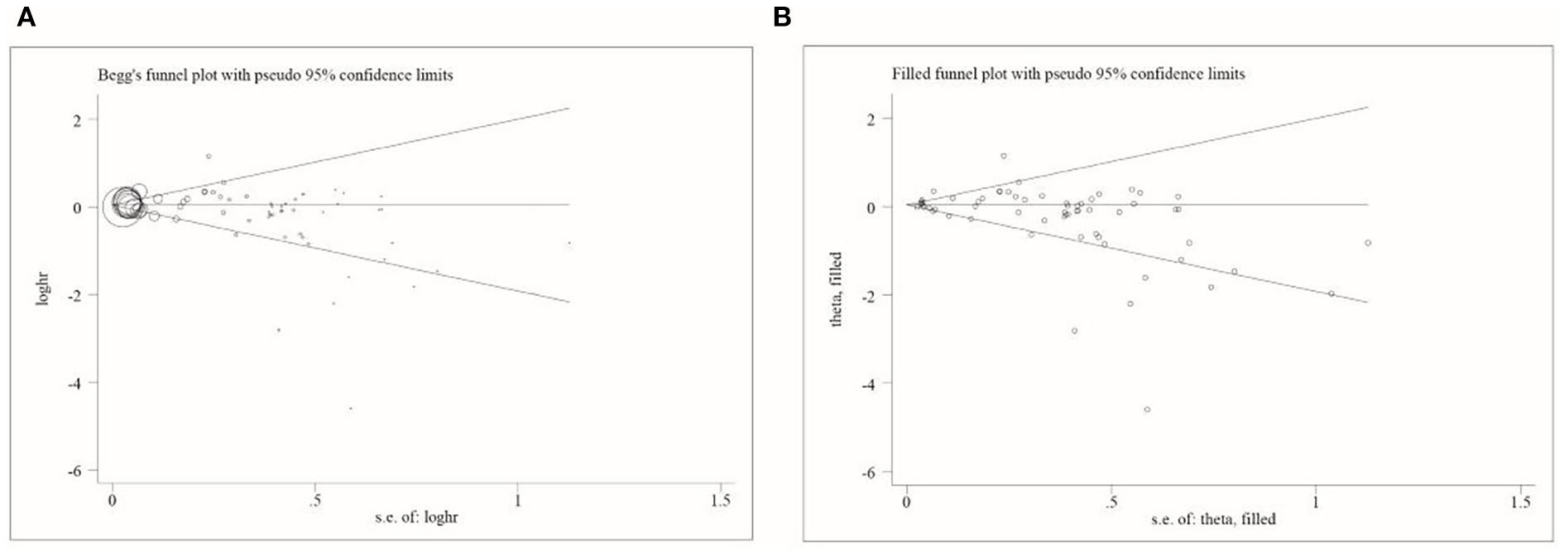
Begg's and filled funnel plots in included studies. **(A)** Begg's funnel plot and **(B)** filled funnel plot.

Publication bias was assessed using Begg's funnel plot and Egger's test. The results showed that Begg's funnel plots were not perfectly symmetrical (Figure 3), Egger's test demonstrated potential publication bias (*P* = 0.014), and no potentially missing study was shown by a further filled funnel plot.

### Sensitive analyses

A sensitivity analysis was performed to assess the influence of individual studies on the pooled ORs. By omitting one study each time, the results revealed that the corresponding pooled ORs did not change, which confirmed the reliability of our results.

### Dose-response analyses

**Figure 4:**
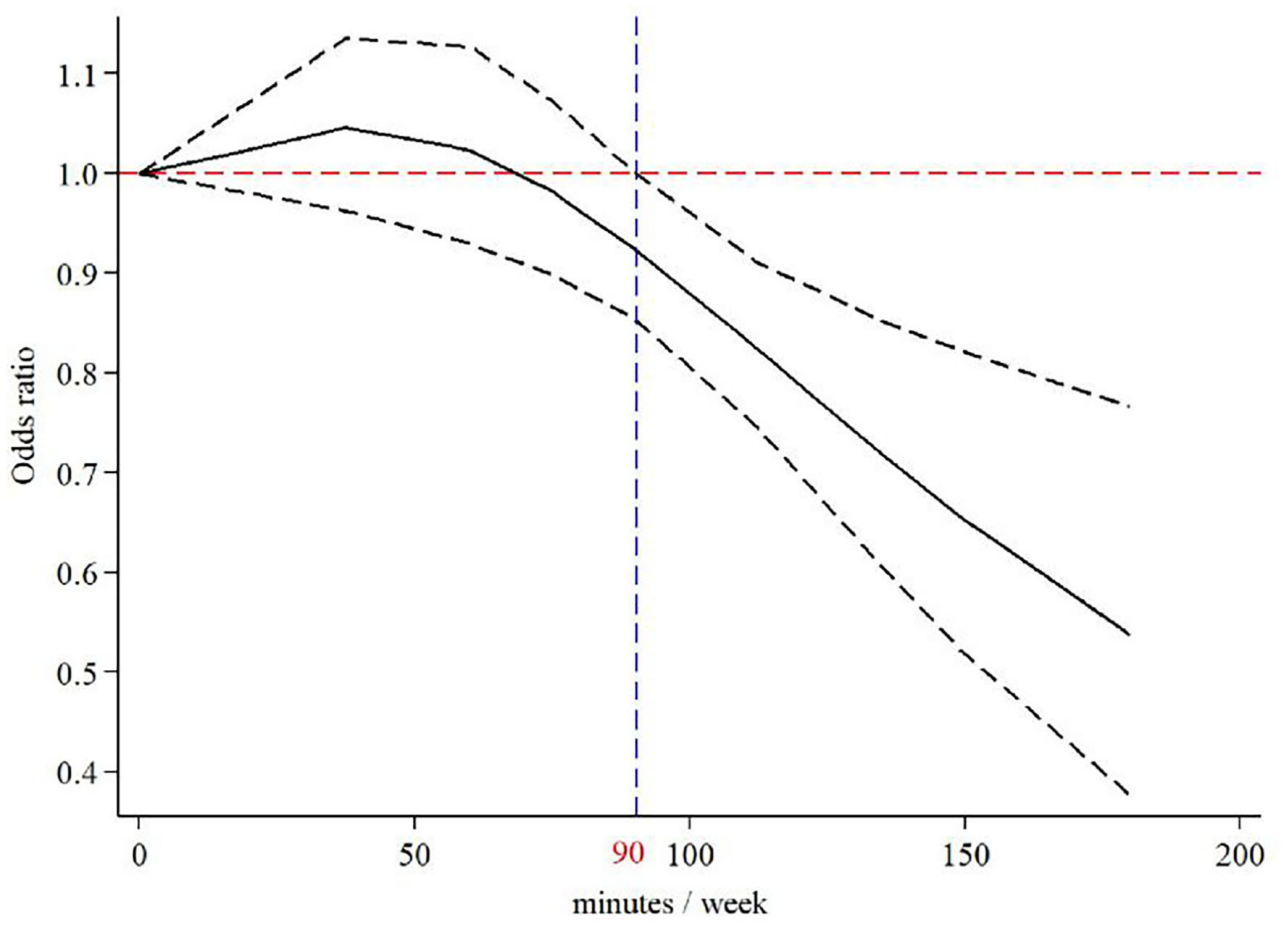
The dose response analysis of physical activity duration and the risk of postpartum depression.

Our dose-response analysis of the relationship between PA duration and the risk of PPD demonstrated an inverted J-shaped trend (Figure 4). The results indicated that longer PA duration decreased the risk of PPD, and when the PA duration reached 90 min per week (adjusted OR = 0.92, 95% CI: 0.85–1.00), an efficient decrease in the risk of PPD was observed (Table 2).

### Subgroup analyses

Subgroup analyses were performed to clarify the cause of between-study heterogeneity (Table 3). Significant heterogeneities were found in the study region, age, gestation age, PA duration, PA type, PA intensity, PA timing, and study type.

**Table 3:** Overall and subgroup analyses of the associations between physical activity and postpartum depression.

**Groups**	**Observations (n)**	**OR (95% CI)**	***P***	***I* ^2^**
Overall analysis				
	63	0.73 (0.61- 0.87)	< 0.001	89.6 %
Subgroup analysis				
By region				
Europe	25	0.85 (0.76–0.95)	0.004	79.1%
America	25	1.12 (0.96–1.30)	0.165	0.3%
Asia	13	1.14 (1.01–1.29)	0.042	87.2%
By age (years old)				
≤ 30	45	1.01 (0.93–1.09)	0.898	78.0%
>30	16	0.92 (0.76– 1.11)	0.364	71.4%
By gestation age (weeks)				
1–12	2	1.21 (0.70–2.12)	0.494	0.0%
13–27	10	0.76 (0.55–1.05)	0.097	23.5%
28–42	14	0.72 (0.39–1.34)	0.303	85.7%
By PA type				
Total	10	1.08 (1.04–1.13)	0.227	23.5%
Work	3	1.05 (0.37–2.97)	0.065	63.5%
Household	11	1.16 (0.89–1.52)	0.986	0.0%
Sports	35	0.89 (0.78–1.00)	< 0.001	84.4%
By PA timing				
Pregnancy period	43	0.99 (0.91–1.06)	0.689	61.9%
After pregnancy	20	0.94 (0.80–1.10)	0.435	88.0%
By study type				
RCT	18	0.53 (0.33–0.86)	0.010	85.6%
Cohort	45	1.04 (0.97–1.10)	0.267	68.8%

PA, physical activity; RCT, randomized control trial; OR, odds ratio; CI, confidence interval.

According to region, our results showed that studies conducted in Europe demonstrated a significant correlation between PA and reduced PPD risk (adjusted OR = 0.85, 95% CI: 0.76–0.95, *P* = 0.004), whereas studies conducted in America (adjusted OR = 1.12, 95% CI: 0.96–1.30, *P* = 0.165) and Asia (adjusted OR = 1.14, 95% CI: 1.01–1.29, *P* = 0.004) displayed a negative association between PA and decreasing PPD risk, with a statistical significance observed in the Asian subgroup.

According to the age of pregnant women, women older than 30 years demonstrated a downward trend of PPD (adjusted OR = 0.92, 95% CI: 0.76–1.11, *P* = 0.364), although the result was not statistically significant. In terms of gestation age of the pregnant participants, an obvious decrease in trend was observed for women in their second trimester (13–27 weeks) (adjusted OR = 0.76, 95% CI: 0.55–1.05, *P* = 0.097) and third trimester (28–42 weeks) (adjusted OR = 0.72, 95% CI: 0.39–1.34, *P* = 0.303).

Next, we divided PA into three major categories, namely, sports, work, and household activities. All kinds of physical exercises, such as yoga, walking, ball games, pilates, and other aerobics workouts, were included in sports activities. Work activities are the amount of muscle- and energy-demanding activities at work. All kinds of housework and childcare were included in household activities. According to our analysis of PA type, sports activities were associated with relieving PPD symptoms (adjusted OR = 0.89, 95% CI: 0.78–1.00, *P* < 0.001). However, work (adjusted OR = 1.05, 95% CI: 0.37–2.97, *P* = 0.065) and household activities (adjusted OR = 1.16, 95% CI: 0.89–1.52, *P* = 0.986) were identified as risk factors of PPD.

According to PA timing, PA during pregnancy (adjusted OR = 0.99, 95% CI: 0.91–1.06, *P* = 0.689) or after pregnancy (adjusted OR = 0.94, 95% CI: 0.80–1.10, *P* = 0.435) demonstrated a downward trend for PPD risk. Study type analysis showed that the results of the RCTs demonstrated a statistically significant association between PA and reduction of PPD risk (adjusted OR = 0.53, 95% CI: 0.33–0.86, *P* = 0.010). In contrast, cohort studies suggested that PA could be regarded as a risk factor for PPD (adjusted OR = 1.04, 95% CI: 0.97–1.10, *P* = 0.267), though the result was not statistically significant.

## Discussion

Based on our literature search and existing knowledge, the present study is the first to investigate the connection between PA and PPD through a dose-response meta-analysis. The main finding of this meta-analysis was that PA was positively associated with a reduced risk of PPD. Synchronously, sensitivity and subgroup analyses revealed a robust association between PA and PPD prevention. Moreover, the dose-response analysis showed a reduced risk of PPD with a longer PA duration and that at least 90 min of PA per week could efficiently prevent PPD.

Subgroup analysis of the study regions indicated that PA was not associated with a reduced risk of PPD in women from Asia, while contradictory results were observed for European women. Previous studies verified that societal differences could influence the incidence of PPD. European and Australian women were observed to have a lower risk of PPD, but women from Asia were osbserved to have higher risks (50, 51). PPD could be caused by diverse factors, including financial problems, cultural differences, and social status. In some Asian countries, such as China, Singapore, and Vietnam, “doing the month” is a tradition for new mothers to recover from the effects of pregnancy. However, such cultural practices might have two-sided effects as, despite providing physical comforts, they can also play a major role in interpersonal conflicts and emotional frustration (52). Existing evidence observed few psychological benefits for new mothers. In Singapore, a prospective cohort study involving 278 new mothers discovered that ~33% of the participants demonstrated negative experiences with such practices during confinement, which significantly contributed to their depression (53). Similarly, a cross-sectional survey including 506 Vietnamese women revealed that, despite following traditional practices, new mothers still suffered from depression symptoms (54). Based on these studies, it is indicated that the occurrence and progression of PPD might be multifactorial.

Different types of PA have different effects on the prevention of PPD. This meta-analysis proved that sports activities played an obvious role in the prevention of PPD, while work and household activities were potential risk factors. It also reported that extensive physical exercise could reduce the risk of depression in pregnant women. An RCT enrolling 57 postpartum women found that yoga was a promising prevention activity for PPD (34). In 2017, a meta-analysis of 13 RCTs demonstrated that aerobic exercise could reduce the risk of PPD (55). Alhough the mechanisms of sports having an impact in reducing PPD risk are not yet fully understood, several hypotheses have been pooled and proposed. From the perspective of biological mechanisms, it is hypothesized that sports could alleviate PPD by enhancing beta-endorphin levels, which are related to improved mood, euphoria, and passion, as well as reduced pain and increased levels of brain neurotransmitters, including dopamine and noradrenaline,which are associated with feelings of satisfaction and euphoria, respectively (56). The neurotrophic factor hypothesis suggests another possible mechanism for PPD reduction. As a necessary neurotrophic factor for normal neural structure development, synaptic transmission maturation, and maintenance, the brain-derived neurotrophic factor (BNDF) is at low levels in patients with depression. However, a study by Aguiar et al. reported that exercise could significantly increase the expression of BDNF mRNA and the level of BDNF in rats and enhance the synaptic plasticity of the hippocampus by increasing the level of BDNF (57). In 2009, Agarwal et al. found that exercise could reduce oxidative stress in rats, suggesting exercise as an influencing factor of oxidative stress, which was a primary cause of depression (58). However, work and household activities were considered strenuous and repetitive, countering the benefits of PA. In contrast, performing involuntary PAs such as work and household activities may be intense; therefore, it is easy to elevate depressive symptoms rather than alleviate them (59, 60).

The majority of the reviewed literature indicated that a greater amount of PA is likely to reduce the risk of depression, concordant with recommendations given in the national physical activity guidelines (i.e., 30 min of moderate-intensity physical activity on most, if not all, days of the week) (61), which indicated that benefits could also be observed for a lower amount of PA, i.e., at least 20–60 min PA/week (62, 63). However, the appropriate duration of PA for pregnant women remains undetermined. Our dose-response analysis revealed that at least 90 min of PA per week, except for household and work activities, could effectively reduce the risk of PPD and improve the mental wellbeing of women.

Previously, Nakamura and Waerden conducted a meta-analysis of 17 articles, including 6 RCTs and 11 observation studies, to assess the effects of PA during pregnancy on PPD (64). They reported that PA was a significant factor in reducing the risk of PPD. Based on their meta-analysis, we expanded the original literature to 23 articles and included only RCT and prospective cohort studies to increase the evidence level of our meta-analysis. To specifically quantify the effects of PA on PPD, the effect size was unified as OR, and a dose-response analysis was conducted, which showed that at least 90 min of PA per week could efficiently prevent PPD. Furthermore, the refinement of the PA type provided a more specific suggestion for new mothers.

There are some limitations to this study. First, only English-language articles were analyzed, possibly leading to a certain level of publication bias. Second, the error of dose-response analysis persisted and was inevitable in secondary analysis; however, the overall reverse J-shaped trend deserves further attention in the association of PA and the risk of PPD. Third, significant heterogeneity was observed in some subgroups, restricting the explanation of pooled effect-size estimates. Finally, since most of the original articles did not report detailed statistics on PA intensity, the relationship between PA intensity and PPD could not be explored in this meta-analysis, and further studies are still needed to fill this gap.

## Conclusion

In summary, PA was identified as a potentially beneficial intervention to reduce the risk of PPD, even though no statistical significance was observed in this meta-analysis. Dose-response analysis between PA and the risk of PPD showed a reduced risk of PPD with a longer PA duration. Furthermore, 90 min of PA per week could efficiently reduce the risk of PPD.

## Data availability statement

The original contributions presented in the study are included in the article/Supplementary material, further inquiries can be directed to the corresponding author/s.

## Author contributions

Conceived and designed the experiments: DW, JL, and XB. Performed the experiments: MY, HC, DC, and CZ. Analyzed the data: MY, FL, XB, and HC. Contributed materials and analysis tools: MY, CZ, DC, FL, and YN. Wrote and revised the article: MY, JL, and DW. All authors read and approved the final manuscript before submission.

## Funding

This work was funded by the Clinical Study on Prevention of Recurrence of Uterine Fibroids After Minimally Invasive Surgery by Integrated Traditional Chinese and Western Medicine (2022-NHLHCRF-LX-02-0116).

## Conflict of interest

The authors declare that the research was conducted in the absence of any commercial or financial relationships that could be construed as a potential conflict of interest.

## Publisher's note

All claims expressed in this article are solely those of the authors and do not necessarily represent those of their affiliated organizations, or those of the publisher, the editors and the reviewers. Any product that may be evaluated in this article, or claim that may be made by its manufacturer, is not guaranteed or endorsed by the publisher.
