# Supplementary material for: Effect of physical activity on prevention of postpartum depression: A dose-response meta-analysis of 186,412 women
Source: Front Psychiatry. 2022 Nov 4;13:984677. doi: 10.3389/fpsyt.2022.984677 (PMC9672674; doi:10.3389/fpsyt.2022.984677)
Supplement: Supplementary file 2 [file Table_2.docx]

| **Search Terms** |
| --- |
| Based on PubMed, Medline, Embase, and Web of Science. |
| (postpartum OR post-partum OR postnatal OR post-natal OR after delivery OR after childbirth OR puerperium) [Title/Abstract], AND (depress* OR mood OR mental health OR depressive disorder* OR gloomy OR blue OR anxiety OR angst OR nervousness OR anxiousness) [Title/Abstract], AND (physical activ* OR exercis* OR physical training OR physical exercis* OR physical fitness OR walking OR running OR jogging OR swimming OR *cycl* OR weight lifting OR yoga OR Tai Ji OR shopping OR working OR sedentary behav* OR clean* OR domestic activit* OR household activit*)[Title/Abstract] |

**Supplementary Table 2. Search terms of this meta-analysis.**
