# Supplementary material for: Effect of physical activity on prevention of postpartum depression: A dose-response meta-analysis of 186,412 women
Source: Front Psychiatry. 2022 Nov 4;13:984677. doi: 10.3389/fpsyt.2022.984677 (PMC9672674; doi:10.3389/fpsyt.2022.984677)
Supplement: Supplementary file 3 [file Table_3.docx]

**Supplementary table 3. Risk of Bias assessment in this meta-analysis.**

| **Author** | Heh | Ko | Da-Costa | Strom | Demissie | SONGØYGARD | Claesson | Demissie | Bershadsky | Mohammadi | Buttner | Szalewska | Daley | Camp-olong | Shakeel | Aguilar | Vargas-Terrones | Coll | Van der Waerden | Özkan | Susukida | Ana | Navas |
| --- | --- | --- | --- | --- | --- | --- | --- | --- | --- | --- | --- | --- | --- | --- | --- | --- | --- | --- | --- | --- | --- | --- | --- |
| **Year** | 2008 | 2008 | 2009 | 2009 | 2011 | 2012 | 2012 | 2013 | 2014 | 2015 | 2015 | 2016 | 2018 | 2018 | 2018 | 2019 | 2019 | 2019 | 2019 | 2020 | 2020 | 2021 | 2021 |
| **1.Bias due to confounding** | | | | | | | | | | | | | | | | | | | | | | | |
| **1.1 Is there potential for confounding of the effect of intervention in this study?** | Yes | No | Yes | Yes | Yes | Yes | Yes | Yes | PY | Yes | PY | Yes | Yes | Yes | Yes | Yes | Yes | Yes | Yes | Yes | Yes | Yes | Yes |
| **1.2 Was the analysis based on splitting participants' follow-up time according to intervention received?** | No | No | No | No | No | No | No | No | No | No | No | No | No | No | No | No | No | No | No | No | No | No | No |
| **1.3 Were intervention discontinuations or switches likely to be related to factors that are prognostic for the outcome?** | Yes | Yes | Yes | Yes | Yes | PY | Yes | Yes | Yes | Yes | Yes | Yes | Yes | Yes | Yes | Yes | Yes | Yes | Yes | Yes | Yes | Yes | Yes |
| **1.4 Did the authors use an appropriate analysis method that controlled for all the important confounding domains?** | No | NI | Yes | Yes | Yes | Yes | PY | Yes | No | No | Yes | No | Yes | Yes | Yes | PN | Yes | Yes | No | No | Yes | Yes | Yes |
| **1.5 Were confounding domains that were controlled for measured validly and reliably by the variables available in this study?** | Yes | Yes | Yes | Yes | Yes | Yes | Yes | Yes | Yes | Yes | Yes | Yes | Yes | Yes | Yes | Yes | Yes | Yes | Yes | Yes | Yes | Yes | Yes |
| **1.6 Did the authors control for any post-intervention variables that could have been affected by the intervention?** | Yes | Yes | No | Yes | Yes | NI | Yes | No | NI | No | No | No | Yes | PY | PY | NI | PY | PY | PY | PN | Yes | Yes | NI |
| **1.7 Did the authors use an appropriate analysis method that controlled for all the important confounding domains and for time-varying confounding?** | PN | PN | Yes | No | Yes | NI | PY | No | No | No | No | No | Yes | No | PY | NI | NI | NI | No | NI | Yes | Yes | NI |
| **1.8 Were confounding domains that were controlled for measured validly and reliably by the variables available in this study?** | Yes | Yes | Yes | Yes | Yes | Yes | Yes | No | NI | No | NI | No | Yes | Yes | Yes | NI | PY | PY | Yes | NI | Yes | Yes | NI |
| **Risk of bias judgement** | Low | Low | Low | Low | Low | Moderate | Low | Moderate | Moderate | Moderate | Moderate | Serious | Low | Low | Low | Moderate | Low | Low | Low | Moderate | Low | Low | Moderate |
| **What is the predicted direction of bias due to confounding?** | FE | FE | FE | FE | FE | FE | FE | FE | FE | FE | FE | Unpredictable | FE | FE | FE | FE | FE | FE | FE | FE | FE | FE | FE |
| **2.Bias in selection of participants into the study** | | | | | | | | | | | | | | | | | | | | | | | |
| **2.1Was selection of participants into the study (or into the analysis) based on participant characteristics observed after the start of intervention?** | No | No | No | No | No | No | No | No | No | No | No | Yes | No | No | No | No | No | No | No | No | No | No | No |
| **2.2 Were the post-intervention variables that influenced selection likely to be associated with intervention?** | Yes | Yes | Yes | Yes | Yes | Yes | Yes | Yes | Yes | Yes | Yes | Yes | Yes | Yes | Yes | Yes | Yes | Yes | Yes | Yes | Yes | Yes | Yes |
| **2.3 Were the post-intervention variables that influenced selection likely to be influenced by the outcome or a cause of the outcome?** | No | No | No | No | No | No | No | No | No | No | No | No | No | No | No | No | No | No | No | No | No | No | No |
| **2.4 Do start of follow-up and start of intervention coincide for most participants?** | Yes | Yes | Yes | No | Yes | Yes | Yes | No | Yes | Yes | Yes | No | Yes | Yes | Yes | Yes | Yes | Yes | No | Yes | No | Yes | Yes |
| **2.5. Were adjustment techniques used that are likely to correct for the presence of selection biases?** | NI | NI | Yes | No | No | Yes | No | No | PN | Yes | Yes | No | Yes | No | No | Yes | Yes | Yes | No | Yes | No | Yes | Yes |
| **Risk of bias judgement** | Low | Low | Low | Low | Low | Low | Low | Low | Low | Low | Low | Moderate | Low | Low | Low | Low | Low | Low | Low | Low | Low | Low | Low |
| **What is the predicted direction of bias due to confounding?** | FE | FE | FE | FE | FE | FE | FE | FE | FE | FE | FE | FE | FE | FE | FE | FE | FE | FE | FE | FE | FE | FE | FE |
| **3.Bias in classification of interventions** | | | | | | | | | | | | | | | | | | | | | | | |
| **3.1 Were intervention groups clearly defined?** | Yes | Yes | Yes | Yes | Yes | Yes | Yes | Yes | Yes | Yes | Yes | Yes | Yes | Yes | Yes | Yes | Yes | Yes | Yes | Yes | Yes | Yes | Yes |
| **3.2 Was the information used to define intervention groups recorded at the start of the intervention?** | Yes | Yes | Yes | Yes | Yes | Yes | Yes | Yes | Yes | Yes | Yes | Yes | Yes | Yes | Yes | Yes | Yes | Yes | Yes | Yes | Yes | Yes | Yes |
| **3.3 Could classification of intervention status have been affected by knowledge of the outcome or risk of the outcome?** | No | No | No | No | No | No | No | No | No | No | No | No | No | No | No | No | No | No | No | No | No | No | No |
| **Risk of bias judgement** | Low | Low | Low | Low | Low | Low | Low | Low | Low | Low | Low | Low | Low | Low | Low | Low | Low | Low | Low | Low | Low | Low | Low |
| **What is the predicted direction of bias due to confounding?** | FE | FE | FE | FE | FE | FE | FE | FE | FE | FE | FE | FE | FE | FE | FE | FE | FE | FE | FE | FE | FE | FE | FE |
| **If your aim for this study is to assess the effect of 1) assignment to intervention, answer questions 4.1 and 4.2; 2) starting and adhering to intervention, answer questions 4.3 to 4.6** | NA | NA | NA | NA | NA | NA | NA | NA | NA | NA | NA | NA | NA | NA | NA | NA | NA | NA | NA | NA | NA | NA | NA |
| **4.Bias due to deviations from intended interventions** | | | | | | | | | | | | | | | | | | | | | | | |
| **4.1 Were there deviations from the intended intervention beyond what would be expected in usual practice?** | Yes | No | No | No | No | No | No | No | No | No | Yes | No | No | No | No | No | No | No | No | No | No | No | No |
| **4.2 Were these deviations from intended intervention unbalanced between groups and likely to have affected the outcome?** | No | No | No | No | No | No | No | No | No | No | No | No | No | No | No | No | No | No | No | No | No | No | No |
| **4.3 Were important co-interventions balanced across intervention groups?** | NA | NA | NA | NA | NA | NA | NA | NA | NA | NA | NA | NA | NA | NA | NA | NA | NA | NA | NA | NA | NA | NA | NA |
| **4.4 Was the intervention implemented successfully for most participants?** | NA | NA | NA | NA | NA | NA | NA | NA | NA | NA | NA | NA | NA | NA | NA | NA | NA | NA | NA | NA | NA | NA | NA |
| **4.5 Did study participants adhere to the assigned intervention regimen?** | NA | NA | NA | NA | NA | NA | NA | NA | NA | NA | NA | NA | NA | NA | NA | NA | NA | NA | NA | NA | NA | NA | NA |
| **4.6 Was an appropriate analysis used to estimate the effect of starting and adhering to the intervention?** | NA | NA | NA | NA | NA | NA | NA | NA | NA | NA | NA | NA | NA | NA | NA | NA | NA | NA | NA | NA | NA | NA | NA |
| **Risk of bias judgement** | Low | Low | Low | Low | Low | Low | Low | Low | Low | Low | Low | Low | Low | Low | Low | Low | Low | Low | Low | Low | Low | Low | Low |
| **What is the predicted direction of bias due to confounding?** | FE | FE | FE | FE | FE | FE | FE | FE | FE | FE | FE | FE | FE | FE | FE | FE | FE | FE | FE | FE | FE | FE | FE |
| **5.Bias due to missing data** | | | | | | | | | | | | | | | | | | | | | | | |
| **5.1 Were outcome data available for all, or nearly all, participants?** | Yes | Yes | Yes | Yes | Yes | Yes | Yes | Yes | Yes | Yes | Yes | Yes | Yes | Yes | Yes | Yes | Yes | Yes | Yes | Yes | Yes | Yes | Yes |
| **5.2 Were participants excluded due to missing data on intervention status?** | NI | PN | No | No | No | No | No | No | No | No | No | No | No | No | No | No | PN | No | No | No | No | No | Yes |
| **5.3 Were participants excluded due to missing data on other variables needed for the analysis?** | NI | PN | No | No | No | No | No | No | No | No | No | No | No | No | No | No | No | No | No | No | No | No | PN |
| **5.4 Are the proportion of participants and reasons for missing data similar across interventions?** | Yes | Yes | Yes | Yes | Yes | Yes | Yes | Yes | Yes | Yes | Yes | Yes | PY | Yes | Yes | Yes | Yes | Yes | Yes | PY | Yes | Yes | Yes |
| **5.5 Is there evidence that results were robust to the presence of missing data?** | No | No | No | No | No | No | No | No | No | No | No | No | No | No | No | No | No | No | No | No | No | No | No |
| **Risk of bias judgement** | Moderate | Low | Low | Low | Low | Low | Low | Low | Low | Low | Low | Low | Low | Low | Low | Low | Low | Low | Low | Low | Low | Low | Low |
| **What is the predicted direction of bias due to confounding?** | Towards null | FE | FE | FE | FE | FE | FE | FE | FE | FE | FE | FE | FE | FE | FE | FE | FE | FE | FE | FE | FE | FE | FE |
| **6.Bias in measurement of outcomes** | | | | | | | | | | | | | | | | | | | | | | | |
| **6.1 Could the outcome measure have been influenced by knowledge of the intervention received?** | No | No | No | No | No | PN | No | No | No | No | No | No | No | No | No | No | No | No | No | No | No | No | No |
| **6.2 Were outcome assessors aware of the intervention received by study participants?** | Yes | Yes | Yes | Yes | Yes | Yes | Yes | Yes | Yes | Yes | Yes | Yes | Yes | Yes | Yes | Yes | Yes | Yes | Yes | Yes | Yes | Yes | Yes |
| **6.3 Were the methods of outcome assessment comparable across intervention groups?** | Yes | Yes | Yes | Yes | Yes | Yes | Yes | Yes | Yes | Yes | Yes | Yes | Yes | Yes | Yes | Yes | Yes | Yes | Yes | Yes | Yes | Yes | Yes |
| **6.4 Were any systematic errors in measurement of the outcome related to intervention received?** | No | PN | No | No | No | No | No | No | No | No | No | No | PN | No | No | NI | NI | PN | No | PN | No | No | No |
| **Risk of bias judgement** | Low | Low | Low | Low | Low | Low | Low | Low | Low | Low | Low | Low | Low | Low | Low | Low | Low | Low | Low | Low | Low | Low | Low |
| **What is the predicted direction of bias due to confounding?** | FE | FE | FE | FE | FE | FE | FE | FE | FE | FE | FE | FE | FE | FE | FE | FE | FE | FE | FE | FE | FE | FE | FE |
| **7.Bias in selection of the reported result** | | | | | | | | | | | | | | | | | | | | | | | |
| **7.1 … multiple outcome measurements within the outcome domain?** | PN | No | No | No | No | No | No | No | No | No | No | No | No | No | No | No | No | No | No | No | No | No | No |
| **7.2 ... multiple analyses of the intervention-outcome relationship?** | PN | No | No | No | No | No | No | No | No | No | No | No | No | No | No | No | No | No | No | No | No | No | No |
| **7.3 ... different subgroups?** | PN | PN | No | No | No | No | No | No | No | No | No | No | No | No | No | No | No | No | No | No | No | No | No |
| **Risk of bias judgement** | Low | Low | Low | Low | Low | Low | Low | Low | Low | Low | Low | Low | Low | Low | Low | Low | Low | Low | Low | Low | Low | Low | Low |
| **What is the predicted direction of bias due to confounding?** | FE | FE | FE | FE | FE | FE | FE | FE | FE | FE | FE | FE | FE | FE | FE | FE | FE | FE | FE | FE | FE | FE | FE |
| **Risk of bias judgement** | Low | Low | Low | Low | Low | Low | Low | Low | Low | Low | Low | Moderate | Low | Low | Low | Low | Low | Low | Low | Low | Low | Low | Low |
| **What is the predicted direction of bias due to confounding?** | FE | FE | FE | FE | FE | FE | FE | FE | FE | FE | FE | FE | FE | FE | FE | FE | FE | FE | FE | FE | FE | FE | FE |

***Abbreviations:*** PY, Probably Yes; PN, Probably No; NI, No Information; FE, Favors experimental; NA, Not available.
